# Supplementary material for: Biophysical Characterization of Adeno-Associated Virus Vectors Using Ion-Exchange Chromatography Coupled to Light Scattering Detectors
Source: Int J Mol Sci. 2022 Oct 22;23(21):12715. doi: 10.3390/ijms232112715 (PMC9655919; doi:10.3390/ijms232112715)
Supplement: Supplementary file 1 [file ijms-23-12715-s001.zip › ijms-1940695-supplementary.pdf]

Supplemental Information

Biophysical Characterization of Adeno-Associated Virus Vectors Using Ion-Exchange Chromatography Coupled to Light Scattering Detectors

Christina Wagner, Bernd Innthaler, Martin Lemmerer, Robert Pletzenauer and Ruth Birner-Gruenberger

Supplementary Table S1: Chromatographic separation method using an AEX column and salt gradient with a flow rate of 0.5 mL/min (B = binding buffer, 20 mM Tris, pH 8.5; E = elution buffer, 20 mM Tris + 120 mM $MgCl_2$, pH 8.5).

Time	Salt gradient	
	%B	%E
0	100	0
2	100	0
27	65	35
28	0	100
32	0	100
32.1	100	0
45	100	0

Supplementary Table S2: Chromatographic separation methods for the determination of the robustness using an AEX column and salt gradients A-D and a flow rate of 0.5 mL/min (B = binding buffer, 20 mM Tris, pH 8.5; E = elution buffer, 20 mM Tris + 120 mM $MgCl_2$, pH 8.5).

Time	Gradient A		Gradient B		Gradient C		Gradient D	
	%B	%E	%B	%E	%B	%E	%B	%E
0	100	0	100	0	100	0	100	0
2	100	0	100	0	100	0	100	0
27	65	35	55	45	45	55	35	65
28	0	100	0	100	0	100	0	100
32	0	100	0	100	0	100	0	100
32.1	100	0	100	0	100	0	100	0
45	100	0	100	0	100	0	100	0

Supplementary Table S3: Chromatographic separation methods using an AEX column and salt gradient (B = binding buffer, 20 mM Tris, pH 8.5; E = elution buffer, 20 mM Tris + 120 mM $MgCl_2$, pH 8.5) or pH gradient (B = binding buffer, 20 mM Tris + 10 mM $MgCl_2$ pH 7; E = elution buffer, 20 mM Tris + 10 mM $MgCl_2$, pH 10) with a flow rate of 0.5 mL/min.

Time	Salt gradient		Time	pH gradient	
	%B	%E		%B	%E
0	100	0	0	100	0
2	100	0	2	100	0
27	65	35	32	0	100
28	0	100	36	0	100
32	0	100	36.1	100	0
32.1	100	0	49	100	0
45	100	0			
